# Effect of Coated Silver Nanoparticles on Cancerous vs. Healthy Cells

**DOI:** 10.1155/2022/1519104

**Published:** 2022-10-08

**Authors:** Liubov Artiukh, Olga Povnitsa, Svitlana Zahorodnia, Calin V. Pop, Nodari Rizun

**Affiliations:** ^1^Department of Virus Reproduction, Zabolotny Institute of Microbiology and Virology, National Academy of Sciences of Ukraine, 154 Akademika Zabolotnoho Street, Kyiv 03143, Ukraine; ^2^Noble Elements LLC/NOBEL, 16 Central Ave, Cheyenne 82001, WY, USA

## Abstract

Unique properties of silver nanoparticles (NPs) ensure their wide applications, in biomedicine; for this reason, it is very important carefully to study the toxicity of such NPs. The influence of silver nanoparticles coated with natural resin (Ag NPs) on the morphological and functional features of healthy BHK-21 and cancerous Hep-2 cells were studied using fluorescence microscopy, MTT, and neutral red assays. Ag NPs induced morphological changes in both cell cultures. The modifications were dose-dependent and more pronounced with an increase in NPs concentration. The IC_50_ value of Ag NPs for Hep-2 cells was found to be 2.19 ± 0.22 *µ*g/mL, whereas for BHK-21 cells it was significantly (5x) higher at 10.92 ± 2.48 *µ*g/mL. The use of NPs at a concentration close to IC_50_ leads to significant increase (up to 40%) in the number of necrotic cells in cancerous cell population and a decrease in the number of mitotic cells (up to 1.3%). In noncancerous cells the cellular parameters were similar to the control cells. These data suggest that the silver nanoparticles coated with natural resin can be potentially used in cancer therapy.

## 1. Introduction

Silver nanoparticles (Ag NPs) are commonly used in many different areas including technology, healthcare, transportation, industrial buildings, information, and communications. Despite the clear advantages of silver NPs in most applications, the toxicological profile and impact on cells existence is mostly unknown [1]. It is common knowledge that silver NPs have the capability of producing reactive oxygen species (ROS), initiating signaling pathways, regulating autophagy, and causing apoptosis. For these reasons, it is important to understand silver toxicity levels and mechanisms of action when designing any silver nanoparticle agent or drug delivery system [2].

With recent development of nanotechnology, silver nanoparticles with known antimicrobial properties have become great candidates for becoming a successful vector in most antibacterial and antiviral applications [3–6].

Several studies so far have used different cell lines to measure the cytotoxicity of Ag NPs and investigate their underlying molecular mechanisms. However, the information gathered by these studies is still not complete. It is known so far that some physical parameters of Ag NPs, such as particle size, shape, surface functionality, and stability along with the dose, mode, and frequency of administration affect cytotoxicity and therefore safety parameters [7].

The purpose of this study was to investigate the influence of silver nanoparticles coated with a natural resin (‘coated nanosilver') on the morphological and functional features of cells.

## 2. Materials and Methods

### 2.1. Characterization of Silver Nanoparticles NPs

The experimental colloidal silver dispersion-containing silver nanoparticles coated with a proprietary natural resins combination was supplied by Noble Elements LLC (Cheyenne, USA). The nominal particle size is 10 ± 1.5 nm and the silver concentration is 2.0 ± 0.05 wt%.

### 2.2. Cell Culture

The Нер-2 cells cancer cells (human laryngeal carcinoma) and BHK-21 (C-13) healthy cells (kidney of Syrian hamster) were obtained from Cell Bank of the Kavetsky Institute of Experimental Pathology, Oncology and Radiobiology of National Academy of Sciences of Ukraine (Kyiv, Ukraine). The cells were cultured in 45% Dulbecco's Modified Eagle's Medium (DMEM, Sigma, USA) and 45% RPMI-1640 (Sigma, USA) with 10% (v/v) fetal bovine serum (FBS, Sigma, USA) and 100 U/mL gentamycin.

### 2.3. Cell Morphology Characterization

Photographs of the interaction of living cells with the silver nanoparticles dispersions of different concentrations were taken over 72 h. The morphological changes were observed using a Carl Zeiss Jena (Germany) light inverted microscope at 70 × magnification.

### 2.4. MTT Assay

Cell viability was assessed using a MTT (3-(4, 5-dimethylthiazol-2-yl)-2, 5-diphenyltetrazolium bromide) assay after NPs exposure, as only viable cells have functional mitochondrial dehydrogenase enzymes which can reduce MTT to formazan [8]. Нер-2 and BHK-21 cells were cultured at a cell density of 1.7 × 10^5^ and 2.2 × 10^5^ cells/mL, respectively, in 96-well plates and incubated for 24 h, 37°C, 5% CO_2_ before the exposure. Cells were later exposed to the Ag NPs in concentrations 40, 20, 10, 5, 2.5, and 1.25 *µ*g/mL. After 72 h of exposure, the cell medium was removed, cells were washed with PBS and 20 *µ*L of MTT solution (5 mg/mL) (Sigma, USA) was added to each well. The cells were incubated for an additional 3 h at 37°C. Next, the MTT solution was removed and 150 *µ*L of 96° ethanol was added to each well. The absorbance of each well was measured in an automatic plate reader Multiskan FC (Thermo Scientific, USA) with a 538 nm test wavelength. The viability (%) of the treated cells was defined as the percentage of absorbance compared to control untreated cells (100% viability). A reduction in viability corresponds to a likelihood of increased cytotoxicity. Percentage of cell viability after exposure to the silver nanoparticle solution was calculated by the following formula [9]:

(1) $$\begin{array}{c} \left(\%\right)\text{cell viability}=\frac{A}{B}\times 100, \end{array}$$

where *A* is the mean optical density of the studied samples at a certain concentration, and *B* is the mean optical density of the control cell samples.

The 50% inhibition concentration (IC_50_) was determined from the dose-response curve and the mean IC_50_ (±S.D.) value of NPs solution was calculated from three independent experiments.

### 2.5. Neutral Red Uptake Assay

In this assay, 1.7 × 10^5^ and 2.2 × 10^5^ cells/mL for Нер-2 cells and BHK-21 cells, respectively, were seeded into 96-well plates and incubated for 24 h to allow for cell attachment, followed by a 72 h treatment with NP suspensions prepared in serum-free cell culture medium (final concentrations of silver NPs solution were 1.25, 2.5, 5, 10, 20, and 40 *µ*g/mL). After treatment, 100 *µ*L of 50 *µ*g/mL neutral red dye (Sigma, USA) was added to each well, and cells were incubated for 3 h, allowing the dye to become permeated inside the acid organelles. Cells were washed with PBS, followed by liberating the assimilated dye by a solvent that consist with 50% v/v ethanol, 1% v/v acetic acid, and 49% v/v deionized water [10].

The released neutral red dye was measured spectrophotometrically (Multiskan FC, Thermo Scientific, USA) at the excitation wavelength of 538 nm. The viability (%) of the treated cells was defined as the percentage of absorbance compared to control untreated cells (100% viability).

### 2.6. Detection of Mitotic and Necrotic Cells through Fluorescence Microscopy

The Нер-2 and BHK-21 cells were grown to form a monolayer in test tubes with strips of cover coat (6 × 22 mm) and then 1 mL/tube of medium with nanoparticles at a concentration of 10, 2 and 0.4 *μ*g/mL was added to the cells. Cells without NPs were used as a control. After 24 h, cells were washed with Hanks solution (BioTestMed, Kyiv, Ukraine) and fixed with 96% ethanol for 1 h. For microscopy, cells were washed with Hanks solution, stained with 0.01% solution of acridine orange (AO) (Sigma, USA) and examined through a fluorescent microscope (AmScope FM690TC, USA) for the presence of necrotic and mitotic cells (using the lens ×40 magnification). In each of the three slides, the number of cells with mitosis and the number of necrotic (pyknotic) cells per 500 counted cells were analyzed. The mitotic index of cells and the percentage of necrotic cells were determined according to the formulas:

(2) $$\begin{array}{c} \text{Mitotic index}\left(\%\right)=\frac{\text{number of cells with mitosis}}{\text{total number of counted cells}}\times 100\text{\%,}\\ \text{Percentage of necrotic cells}=\frac{\text{number of necrotic cells}}{\text{total number of counted cells}}\times 100\text{\%}. \end{array}$$

The changes in mitotic activity and the number of necrotic cells were calculated upon the introduction of different concentrations of NPs in comparison with cells not treated with NPs.

### 2.7. Statistical Analysis

The data from all cytotoxicity experiments were expressed as the arithmetic mean ± standard deviation (SD) and were statistically analyzed by MS Excel. A *p* value lower than 0.05 was considered statistically significant.

## 3. Results

### 3.1. Cell Viability

**Figure 1:**
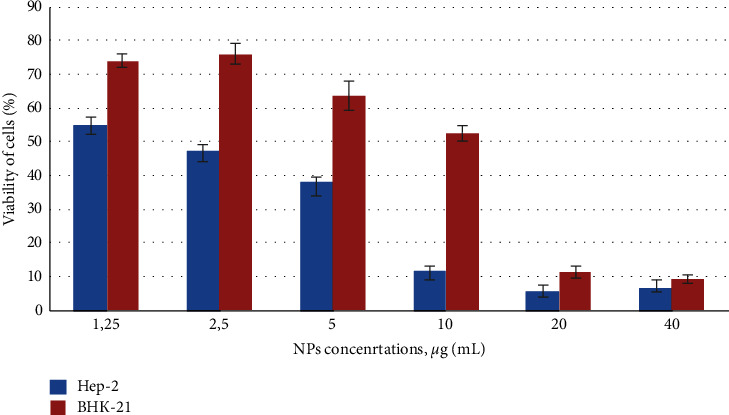
Influence of colloidal-coated silver on viability and mitochondrial activity of cells. Нер-2 and BHK-21 cells growth curves after 72 h exposure with NPs were monitored by colorimetric MTT assay. Control untreated cells − 100% viability. Values represent the mean ± S.D. for three independent experiments. Statistically significant difference between the growth inhibition effect was *p* < 0.05.

MTT is the most commonly used tetrazolium salt for *in vitro* toxicity assessment of nanoparticles. It was performed after 72 hours' cells incubation with various NP concentrations. With the increase of Ag NPs concentration, a significant decrease of cell Hep-2 viability was observed in the 2.5–40 *μ*g/mL range (Figure 1). The cell survival rate decreases were from 48% to 5–10% of the control cells, indicating severe cell damage to cancer cells (abnormal change in cancer cells size, shrinkage, and rounded appearance of cells). In contrast, Ag NPs exhibited a significantly lower effect on the healthy, noncancerous cells ВНК-21 as at a concentration of 5 *μ*g/mL the cell viability was 63% and at 10 *μ*g/mL 52% (Figure 1). It is important to notice that at 2.5 *μ*g/mL of silver the viability of healthy cells was close to 80% while the viability of cancer cells was less than 50%.

**Figure 2:**
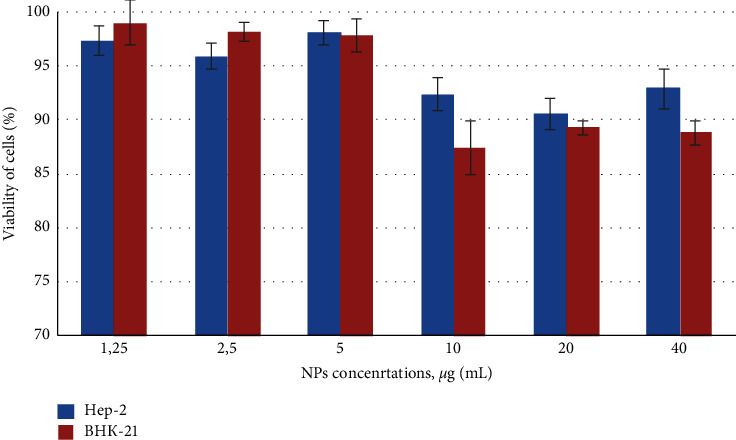
Effect of colloidal coated silver on lysosomal activity of cells. Cytotoxicity of NPs was assessed after 72 h exposure on Нер-2 and BHK-21 cells, evaluated by neutral red uptake (NRU) assay. Control untreated cells − 100% viability. Error bars depict the standard error of the mean. Results show the same lack of effect of nanosilver on cell viability (*p* < 0.05).

Lysosomal integrity in NPs exposed Нер-2 and BHK-21 cells was assessed by neutral red uptake (NRU) assay [10]. This assay is based on the ability of viable cells to maintain an acidic pH inside lysosomes (an ATP-dependent process). The weak cationic neutral red dye penetrates cell membranes and concentrates in the acid environment of the lysosomes. The quantity of the neutral red dye retained in the lysosomes can be measured spectrophotometrically. With the NRU assay, we did not detect any significant lysosomal integrity changes in Hep-2 and BHK-21 cells treated by NPs (Figure 2), as was noticed a small decrease in intensity of neutral red (approximately 12%, statistically insignificant).

### 3.2. Morphologic Changes

**Figure 3:**
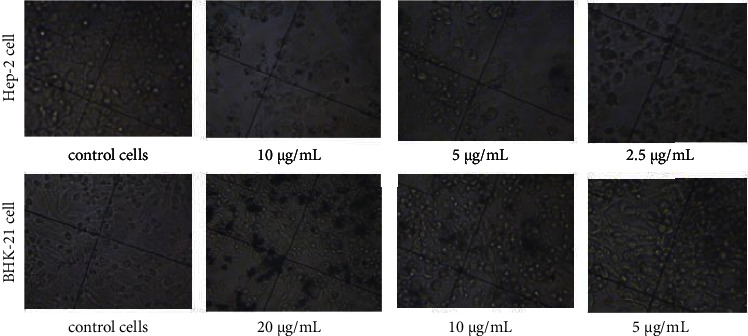
Morphology changes of cells before and after adding colloidal-coated silver. Inverted microscope pictures of cells in the presence of varying concentrations of Ag NPs at 72 h exposure time (70× magnification).

Following NPs exposure at various doses, the cell morphology was analyzed and documented using an inverted microscope. Changes in the morphology of Hep-2 and BHK-21 cells exposed the Ag NPs were observed as compared to unexposed cells (Figure 3).

In the absence of NPs, at 72 h the cells displayed typical polygonal (Нер-2) and elongated (BHK-21) shape and intact morphology, whereas in the presence of NPs, changes in the cell morphology were observed. Even at 2.5 *μ*g/mL concentration of Ag NPs, changes in the Hep-2 cancer cells shape were visible (Figure 3). It was seen that with an increase in silver concentration the proportion of cell contraction and deformation also increased. A decrease in the cell density is also evident with an increase in NP concentration (Figure 3). An increased number of detached Hep-2 cells with spherical morphology was seen at 2.5–20 *μ*g/mL of Ag NPs exposure. It should be noted that at high concentrations the NPs formed aggregates in or outside the cell.

**Figure 4:**
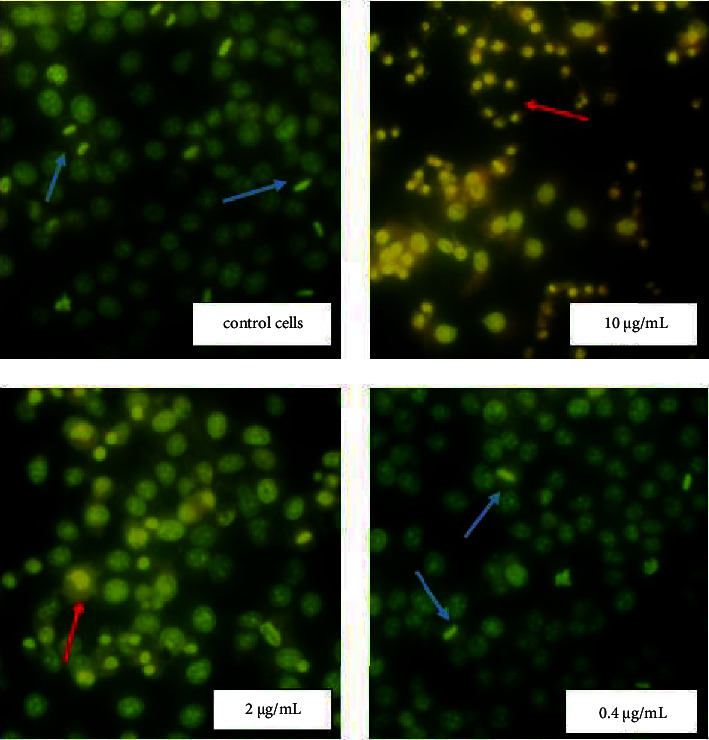
Morphological changes of Hep-2 cells exposure Ag NPs. The cells morphology after Ag NPs treatment were analyzed by fluorescent microscopy after staining with acridine orange. NPs at concentrations 10 and 2 *μ*g/mL lead to significant morphological changes in cancer Hep-2 cells such as pyknosis of nuclear chromatin and loses of the cytoplasm contents. Red arrows show necrotic cells and blue arrows − mitotic cells (280× magnification).

**Figure 5:**
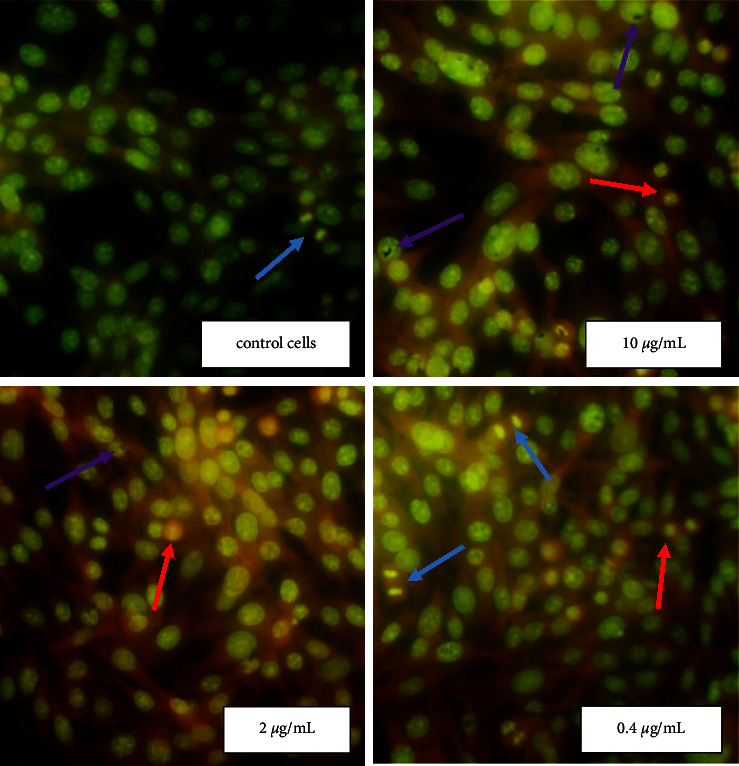
Morphological changes of BHK-21 cells exposure Ag NPs. The cells morphology after Ag NPs treatment were analyzed by fluorescent microscopy after staining with acridine orange. In noncancerous cell BHK-21, regardless of nanoparticles concentrations the cellular parameters were similar to the untreated control cells. Red arrows show necrotic cells, and blue arrows − mitotic cells, while purple arrows show conglomerates of NPs (280× magnification).

Acridine orange (AO) is a heterocyclic organic compound which is a versatile nucleic acid selective fluorescence dye used to stain acidic vacuoles, RNA and DNA in living cells [11]. To determine the mitotic index of cells and the percentage of necrotic cells, the fixed tissue was stained with 0.01% acridine orange and examined through a fluorescent microscope. In controls Hep-2 and BHK-21 cells (untreated NPs), the nucleolus and cytoplasm were orange, and nuclear chromatin was in form of uniformly distributed small bright green granules (Figures 4 and 5).

Homogeneous orange glow characteristic of pyknotic cells and green chromatin glow during cell mitosis were sometimes detected. It should be noted that during necrosis, the cells initially had a shrunken nucleus with absent nuclear demarcation. These cells ultimately lacked cellular RNA and possessed a smooth, shrunken, and bright nucleus (Figures 4 and 5). Ag NPs in concentrations 10 and 2 *μ*g/mL (IC_50_ value) induced distinct morphological changes of Hep-2 cancer cells stained with AO (Figure 4). At the use of NPs in concentration 2 *μ*g/mL, a significant increase in the number of necrotic cells in the Hep-2 population (up to 40%) and a decrease in the number of mitotic cells up to 1.3% were found (Table 1). However, no changes in the morphology and viability of the Hep-2 cell population were detected at a NPs concentration of 0.4 *μ*g/mL (Figure 4). When using NPs at a concentration close to IC_50_ value (10 *μ*g/mL) the number of necrotic cells in the healthy BHK-21 population was similar to the control untreated NPs cells (Figure 5 and Table 1). However, an increase in the mitotic index of cells up to 2.3% (statistically insignificant) compared with the control cells was recorded.

## 4. Discussion

It is known, that the physicochemical and structural features of Ag NPs play an important role in their interaction with cells [6]. Different properties can trigger different levels of potential toxicity effects. For this reason, the physicochemical properties of Ag NPs are fundamental parameters to be considered in risk assessments and studies of various effects on macro-organisms. Silver NPs can affect the metabolism of a living cell, potentially disrupting its natural evolution course, through various mechanisms, including the formation of free radicals. Therefore, recently, a fairly large number of experimental results have been published on cell culture toxicity [12].

The cell viability assay is an important method of toxicology analysis. It can explain cellular response to various toxic materials and it can provide information on cellular survival and metabolic activities [13]. Previous studies reported that cytotoxicity on HeLa cell lines increased with an increase in the concentration of Ag NPs [14]. The enhanced cytotoxic activity of Ag NPs on MCF7 cells observed due to decreased viability and proliferation of cells, and apoptosis (induced programmed cell death) [15]. Molina et al. reported that colloidal silver induced a dose-dependent cytotoxic effect on breast cancer cells [16]. In support of these studies, we confirmed that Ag NPs induced some morphological changes in Hep-2 cells and BHK-21 cells. The microscopic evaluation of cells exposed to Ag NPs resulted in the loss of the characteristic monolayer of these cells. Recently, Liao et al. have reported dose, time, and size dependency of the Ag NPs mediated cytotoxicity, mainly for particles smaller than 10 nm [17].

According to our results, the cell lines exposed to silver NPs lost their adherent ability, as evidenced by the complete or partial breakdown of the monolayer integrity, as well as by the shrinking cells to more granular and rounder. Cells may adhere to each other and to the extracellular matrix through certain cell-surface and membrane proteins called cell adhesion molecules (CAMs). Silver ions or nanoparticles can attach to these proteins thus influencing the cell adherence. Cell adherence phenomenon, as well as multiple cell adhesion molecules, may participate in intercellular and extracellular matrix interactions in cancer [18]. Cancer progression is a complex process in which some adhesion molecules play an essential role in the development of recurrent, invasive, and distant metastasis.

It was shown that the colloidal coated silver concentrate manifests a functional dose-dependent cytotoxic effect against cancerous cell lines. Relatively low concentrations (2.19 ± 0.22 *µ*g/mL) of Ag NPs inhibited 50% of cancerous Hep-2. In contrast, it took a five times higher concentration (10.92 ± 2.48 *µ*g/mL) of silver nanoparticles to obtain a similar effect. The reduced viability with silver exposure of human adenocarcinoma cell lines could be attributed to the apoptotic or necrotic effect induced by the silver nanoparticles.

Many previous studies demonstrated that Ag NP-induced cytotoxicity was mainly caused by oxidative stress caused when the generation of ROS exceeds the cell's antioxidant capacity [19]. However, other mechanisms for cytotoxicity of Ag NPs such as modification of the mitochondrial membrane potential, DNA fragmentation, leakage of lactate dehydrogenase, activation of apoptotic caspases, and nuclear fragmentation were also proposed [9, 20].

The lysosome is an essential organelle inside the cell which plays a role in nutrient recycling and energy metabolism in response to external stress [21]. At present, many studies reported that several engineered nanomaterials at a high dose could result in lysosomal dysfunction and cause autophagy. The result of the neutral red assay showed that Ag NPs do not cause any significant lysosomal integrity changes in Hep-2 and BHK-21 cells.

There are conflicting examples in the literature about necrotic effects of NPs. This is because most reports only studied lost cell viability without focusing on the exact model of cell death, and also because sometimes apoptosis and secondary necrosis interfered with results, leading to incorrect interpretation [1, 22]. The specific cell response to the presence of NPs is complex and determined by many diverse factors. For example, low concentrations of silver NPs may or may not induce apoptosis, whereas necrosis alone is triggered at higher concentrations [23]. Moreover, the exposure time of the Ag NPs and type of cells (such as skin, fibro-sarcoma, or testicular carcinoma) dictates the mode of cell death (apoptosis or necrosis) [24, 25]. High dose Ag NP exposure causes a reduction in antioxidant enzymes such as GSH, and results in elevated levels of intracellular ROS and the consequent elevated expression of ROS-responsive genes, lipid peroxidation, and ultimately, DNA damage, necrosis, and apoptosis [1]. The ability of coated silver nanoparticles (Ag NP) to inhibit and destroy cancer cells at certain doses where normal cells are not affected makes coated silver nanoparticle a great candidate for consideration in future cancer therapy.

It is known that in the late stages of necrosis, the cytoplasm loses contents and takes on a homogeneous eosinophilic appearance, shows irregularities in the membrane of cytoplasmic organelles, mitochondrial swelling, increased matrix density, the formation of vacuoles, and the deposit of calcium phosphates. At the nuclear level, chromatin patterns are seen with pyknosis (chromatic condensation), karyorrhexis (nuclear fragmentation), and karyolysis (complete chromatin disruption). Using dye AO and fluorescence microscopy it was detected that Hep-2 and BHK-21 cells contained predominantly green nuclei and a red cytoplasmic consistent with RNA localization. Necrotic cells are characterized as completely lacking cellular RNA, but having a bright nucleus. We found that NPs at a concentration close to IC_50_ lead to significant morphological changes in cancer Hep-2 cells and an increase in the number of necrotic cells (by up to 40%) compared with control cells. At the same time, no changes in the morphology and viability of human adenocarcinoma cells treated with NPs in the concentration of 0.4 *μ*g/mL were detected. More specifically, a concentration of 0.4 *µ*g/mL had no effect on cancer cells while the increase in the silver concentration to 2.5 *µ*g/mL destroyed 40% of cancer cells. Many previous studies have revealed the effect of Ag NPs on cellular biological function while exposed to much higher doses in cancer treatment (against cervical cancer, breast cancer, lung cancer, hepatocellular carcinoma, nasopharyngeal carcinoma, hepatocellular carcinoma, glioblastoma, colorectal adenocarcinoma, and prostate carcinoma). In general, the smaller the particle size, the higher the active surface and biological activity [26].

Another factor that can modulate the effect of silver nanoparticles is their ability to aggregate [6]. The degree of aggregation varies depending on many factors like the pH, the electrolyte or salt content, and the protein composition in the culture medium [27]. The natural resin coating of the silver nanoparticles used in this study is designed to greatly diminish the degree of particle aggregation However, we found that colloidal-coated silver at higher concentrations may form aggregates in the cytoplasm and cellular nuclei.

## 5. Conclusions

This study confirms that silver nanoparticles coated with a natural resin layer induces morphological changes in cancerous Hep-2 and in healthy BHK-21 cells. The changes in the cell morphology were dose dependent and more pronounced with an increase in silver NPs concentration. It was found that the viability results were dependent on the type of cells used. The 50% cytotoxic concentration (IC_50_ value) of Ag NPs for cancerous cells was 2.19 *µ*g/mL, whereas in noncancerous cell line, it was significantly (5x) higher at 10.92 *µ*g/mL. At the same time, the result of the neutral red assay showed that Ag NPs do not cause any significant lysosomal integrity changes in healthy or cancerous cells.

It should also be noted that the use of coated Ag NPs at a concentration close to IC_50_ leads to a significant increase in the number of necrotic cells in cancerous cell population (up to 40%) and a decrease in the number of mitotic cells up to 1.3%. This shows that the proliferation rate of cancer cells actually slows down. At a silver concentration of 2.5 *µ*g/mL, the viability of healthy cells was close to 80% while the viability of cancer cells was less than 50%.

In conclusion, it was shown that the colloidal-coated silver concentrate manifests a functional dose-dependent cytotoxic effect against cancerous cell lines, and in concentration of 2.5 *µ*g/mL destroyed 40% of cancer cells. At the same time, in noncancerous cell lines, the cellular parameters were similar to the control cells which were untreated.

The abovementioned findings show that the coated silver nanoparticles inhibit and destroy cancer cells at certain doses where normal cells are not affected. This strongly suggests that silver nanoparticles coated with natural resins could be a great candidate for consideration in future cancer therapy.

## Figures and Tables

**Table 1:** Cytomorphological study of Нер-2 and ВНК-21 cells treated with silver NPs.

	Нер-2	+NPs 2 *μ*g/mL	+NPs 0.4 *μ*g/mL
Average number of total counted cells	614 ± 24	504 ± 15	538 ± 11
Average number of mitotic cells	28 ± 3	7 ± 2	26 ± 6
Average number of necrotic cells	10 ± 1	202 ± 9	10 ± 2
Mitotic index, %	4.6	1.3	4.9
Necrotic cells, %	1.7	40.1	1.9

	ВНК-21	+NPs 10 *μ*g/mL	+NPs 2 *μ*g/mL
Average number of total counted cells	555 ± 14	568 ± 10	532 ± 13
Average number of mitotic cells	8 ± 2	13 ± 2	10 ± 2
Average number of necrotic cells	25 ± 4	24 ± 3	23 ± 4
Mitotic index, %	1.4	2.3	1.9
Necrotic cells, %	4.4	4.3	4.3

## Data Availability

All data used to support the findings of this study are included within the article and available from the corresponding author upon request.
